# Magnetic Resonance Imaging Image Segmentation under Edge Detection Intelligent Algorithm in Diagnosis of Surgical Wrist Joint Injuries

**DOI:** 10.1155/2021/6891120

**Published:** 2021-10-01

**Authors:** Zhongyi Li, Xi Ji

**Affiliations:** ^1^Department of Second Hand Surgery, Affiliated Central Hospital of Shenyang Medical College, Shenyang 110000, Liaoning, China; ^2^Department of Foot and Ankle Surgery, Affiliated Central Hospital of Shenyang Medical College, Shenyang 110000, Liaoning, China

## Abstract

**Background:**

Wrist joint injury refers to the injury of the wrist joint caused by excessive stretching of the ligaments and joint capsules around the joint caused by indirect violence. The tissue structure of the wrist joint is complex, and the clinical diagnosis effect is poor.

**Methods:**

The purpose of this study was to improve the diagnostic accuracy of wrist joint injuries and provide evidence for imaging analysis and automatic diagnosis of lesions in patients with wrist joint injuries. The Canny algorithm was adopted to extract the edge features of the patient's magnetic resonance imaging (MRI) image, and the particle swarm optimization-support vector machine (PSO-SVM) algorithm was applied to segment the lesion. The image processing effect of the algorithm was evaluated by taking peak signal to noise ratio (PSNR), mean square error (MSE), figure of merit (FOM), and structural similarity (SSIM) as indicators. The accuracy, sensitivity, specificity, and Dice similarity coefficient of the algorithm were analyzed to evaluate the diagnostic accuracy in WJI.

**Results:**

Compared with the Gradient Vector Flo (GVF) algorithm and the Elastic Automatic Region Growing (ERG) algorithm, the edge stability of the PSO-SVM algorithm was stable above 0.9. After the quality of images processed using different algorithms was analyzed, it was found that the PSNR of the PSO-SVM algorithm was 26.891 ± 5.331 dB, the MSE was 0.0014 ± 0.0003, the FOM was 0.8832 ± 0.0957, and the SSIM was 0.9032 ± 0.0807. The four indicators were all much better than those of the GVF algorithm and the EARG algorithm, showing statistically obvious differences (*P* < 0.05). Analysis on diagnostic accuracy of different algorithms for WJI suggested that the diagnostic accuracy of the PSO-SVM algorithm was 0.9413, the sensitivity was 0.9129, the specificity was 0.9088, and the Dice similarity coefficient was 0.8715. The four indicators all showed statistically great difference compared with those of the GVF algorithm and the EARG algorithm (*P* < 0.05).

**Conclusions:**

The PSO-SVM algorithm showed excellent edge detection performance and higher accuracy in the diagnosis of WJI, which can assist clinicians in the clinical auxiliary diagnosis of WJI.

## 1. Introduction

Joint is an indirect connection form among human bones and is an important part of the motion system [1]. Joint can be divided into three categories: movable joints, half joints, and immobile joints according to the nature and activity of their connected tissues. Most of the bone connections in the human body belong to movable joints, such as wrist joints, elbow joints, and knee joints [2, 3]. The wrist joint is a typical elliptical joint, composed of the proximal ulna, radius, five distal metacarpal bones, and eight carpal bones. The wrist bones are short bones with different shapes and small sizes, which are manifested by their own structural complexity and functional flexibility [4, 5]. The wrist joint is one of the most important and most complex joints in the human body [6] and plays an important role in people's daily activities because it is easily damaged in people's daily life.

Wrist joint injury (WJI) includes ligament, synovial damage, articular cartilage, bone cortical damage, and bone marrow lesions caused by trauma [7], which has a greater impact on the patient's hand function. After the injury occurs, fixed treatment or surgical treatment is usually used. If the treatment is improper, it may cause delayed healing or abnormal healing of the injured site [8]. X-ray, computed tomography (CT), magnetic resonance imaging (MRI), and other imaging examination methods play important roles in the diagnosis of wrist injuries. X-ray and CT are the main examination methods of WJI at present. X-rays are two-dimensional planar projections, which require clinicians to have advanced spatial judgment ability. When the wrist joint is seriously injured, the structural disorder causes the anatomical structure to be more complicated, and it is difficult to obtain accurate examination results based on X-ray films [9, 10]. Although CT examination is not affected by wrist overlap and radial fossa depression, the thickness of conventional CT can easily lead to missed diagnosis of axial fracture line, and it is also difficult to distinguish carpal displacement [11]. MRI shows nonradiation, multiplane, multiparameter imaging, and good soft tissue contrast [12] can provide a lot of relevant lesion information and can more accurately determine the damage mechanism. However, some patients cannot tolerate long-term MRI scans, or show poor cooperation due to pain and other reasons, resulting in interferences such as noise and motion artifacts in MRI images [13], making it difficult to obtain the expected diagnosis when diagnosing WJI patients effect.

Traditional WJI diagnosis usually requires clinicians to analyze and judge one by one based on the patient's MRI images, but this diagnosis method is subjective, and the judgment result will inevitably affect clinical diagnosis and treatment to a certain extent [14]. With the continuous development of artificial intelligence (AI), automatic segmentation algorithms have been rapidly developed in the automatic detection and segmentation of medical images [15]. Commonly used medical image segmentation methods are divided into three types: threshold-based segmentation, edge-based segmentation, and region-based segmentation. Edge-based segmentation methods are generally implemented by difference operators. The edge detection effect of medical images needs to consider edge continuity, closure, and edge positioning accuracy. The current research status of edge detection can be divided into two categories: classic edge detection algorithms and new edge detection technologies [16]. Classic edge detection algorithms include Roberts operator, Sobel operator, Laplacian operator, and Canny operator. As the core operator of edge detection, Canny operator has good antinoise performance and accurate edge positioning function, but the edge detection performance of Canny operator will decrease when applied to complex images [17]. Cao et al. [18] improved the edge detection performance by introducing the Otsu algorithm to optimize the double threshold of the Canny operator and increased the image processing speed by about 3.4 times. However, the wrist joint tissue structure is complex, and the artifacts and gray unevenness caused by MRI scanning are still difficult for automatic segmentation [19]. Based on the above reasons, an automatic segmentation method for wrist MRI images was proposed in this study based on the edge detection algorithm, which improved the diagnostic accuracy of WJI. This study aimed to provide evidence for the imaging analysis and automatic diagnosis of WJI patients.

## 2. Materials and Methods

### 2.1. Research Subjects

In this study, 160 WJI patients who admitted in hospital from August 2018 to April 2021 were selected as the research subjects, including 73 males and 87 females, aged 21–68 years old (with an average age of 45.1 ± 18.8 years old). This study had been approved by the ethics committee of hospital. All patients and their families were aware of the study and had signed the informed consent forms.

The inclusion criteria were defined as follows: patients ≥18 years old, and patients had a history of wrist injury within 1 week.

The exclusion criteria were defined as follows: patient with cognitive impairment; patients had contraindications to MRI scanning; and patients had wrist joint infection, tumor, or pathological fracture caused by other diseases.

### 2.2. Scanning Method

All patients underwent MRI scans in the wrist joints using superconducting MRI machines. The method was as follows: the scanning slice thickness was 3 mm, and the slice spacing was 2 mm. The sagittal and coronal spin echo sequence (T1WI) and the transverse short-time reversal sequence (STIR) were used to scan the patients during the scanning process. Among them, T1WI sequence (time of repetition (TR) and time of echo (TE) were 545 and 120, respectively; ETL5, field of view (FOV) was 14 × 14 cm, and matrix was 512 × 512); STIR sequence (TR and TE were 3400 and 30, respectively; ETL12, FOV was 10 × 10 cm, and the matrix was 576 × 576); and coronal STIR sequence (TR was 3000, TE was 30, ETL12, FOV was 10 × 10 cm, and matrix was 512 × 512). After the scan, comprehensive analysis of the patient's MRI image is performed to obtain the diagnosis result.

### 2.3. MRI Image Segmentation Method Based on Edge Detection Algorithm

When a patient's MRI image was segmented, the amplitude of the radio frequency signal in MRI represented the image intensity of each image. On an image, there was a unique measurement image at each position, which was called a scalar image. The measured image with more than one images was called a vector image. MRI was to obtain images in a discrete space, and image segmentation was to divide an image into regions that were not overlapped with each other. *P* was adopted to denote the area to which the original image belonged, then *P* should meet the following conditions:

(1) $$\begin{array}{c} P=\overset{K}{\underset{k=1}{\cup }}C_{k}. \end{array}$$

In the above equation, *K* represented the number of clusters, *C*_*k*_ referred to pixel clustering, and pixel clustering was to extract a certain region of interest in the image. The edge detection algorithm was a partial image segmentation method. Its purpose was to use the feature of the extreme difference between the grayscale of the edge point of the image and the adjacent grayscale, and the image edge can be obtained by solving maximum values of the first-order and second-order degrees of the image in the horizontal and vertical directions [20]. When edge detection was performed, it had to use the edge detection operator to detect all possible edge points in the image. In this study, the Canny edge detection algorithm was adopted to extract the edge points of the image. The Canny algorithm could detect the strong and weak edges of the image through high and low thresholds. When weak edges were connected to strong edges, only weak edges were included in the output. The Canny algorithm had three edge detection criteria: the signal-to-noise ratio (SNR) principle, the positioning accuracy principle, and the single-edge response principle [21]. The function expressions of the above three edge detection criteria were as follows:

(2) $$\begin{array}{c} \text{SNR}=\frac{\left|\int_{-\varpi }^{+\varpi }E\left(-x\right)r\left(x\right)\text{d}x\right|}{n_{0}\sqrt{\int_{-\varpi }^{+\varpi }r^{2}\left(x\right)\text{d}x}}, \end{array}$$

(3) $$\begin{array}{c} \text{Loc}=\frac{\left|\int_{-\varpi }^{+\varpi }E^{\prime }\left(-x\right)r^{\prime }\left(x\right)\text{d}x\right|}{n_{0}\sqrt{\int_{-\varpi }^{+\varpi }{r^{\prime }}^{2}\left(x\right)\text{d}x}}, \end{array}$$

(4) $$\begin{array}{c} D\left(r^{\prime }\right)=\sqrt{\frac{\int_{-\infty }^{+\infty }{r^{\prime }}^{2}\left(x\right)\text{d}x}{\int_{-\varpi }^{+\varpi }r\left(x\right)\text{d}x}}. \end{array}$$

In equations (2)–(4) above, *E*(−*x*) referred to the image edge coefficient, *ϖ* was the filter window width, *r*(*x*) represented the impulse response, *n*_*0*_ was the Gaussian noise mean square error, *E′*(−*x*) was the first derivative of *E(*−*x)*, and *r'*(*x*) referred to the first derivative of *r*(*x*).

A two-dimensional Gaussian function was adopted to smooth the original image, and the obtained smoothed image function expression was as follows:

(5) $$\begin{array}{c} P\left(x,y\right)=P_{0}\left(x,y\right)\times G\left(x,y\right). \end{array}$$

In the above equation, *P*_0_(*x*, *y*) represented the original input image, *G*(*x*, *y*) was the two-dimensional Gaussian function, and *P*(*x*, *y*) referred to the smoothed image. The function expression of *G*(*x*, *y*) was as follows:

(6) $$\begin{array}{c} G\left(x,y\right)=\frac{1}{2\pi \sigma^{2}}\exp\left(\frac{y^{2}-x^{2}}{2\sigma^{2}}\right). \end{array}$$

In the above equation, *σ* represented the distribution parameter of the two-dimensional Gaussian function. The larger the *σ* value, the higher the image SNR, the lower the edge detection accuracy, and the smoother the image. After the image smoothing was over, the magnitude and direction of the image gradient of *P*(*x*, *y*) were calculated, in which the derivatives in the *x* and *y* directions were calculated, respectively (as shown in the following equations):

(7) $$\begin{array}{c} \nabla_{x}P\left(x,y\right)=\frac{\partial P\left(x,y\right)}{\partial x},\\ \nabla_{y}P\left(x,y\right)=\frac{\partial P\left(x,y\right)}{\partial y}. \end{array}$$

On the basis of obtaining the partial derivatives in the *x* and *y* directions, the function expressions for the gradient magnitude and direction of the smoothed image were as follows:

(8) $$\begin{array}{c} D\left(x,y\right)=\sqrt{{\left(\nabla_{x}P^{\prime }\left(x,y\right)\right)}^{2}+{\left(\nabla_{y}P^{\prime }\left(x,y\right)\right)}^{2}}, \end{array}$$

(9) $$\begin{array}{c} \theta \left(x,y\right)=\arctan\frac{\nabla_{y}P^{\prime }\left(x,y\right)}{\nabla_{x}P^{\prime }\left(x,y\right)}. \end{array}$$

In equations (8) and (9), *D*(*x*, *y*) represented the gradient magnitude of the smooth image, *P*′(*x*, *y*) represented the smooth image, and *θ*(*x*, *y*) referred to the angle between the gradient magnitude vector and the *x*-axis. In order to ensure that the image edge detection result was one-pixel width, the double threshold method was adopted to set the high and low thresholds, and the hysteresis binarization was applied for edge connection to avoid the edge loss phenomenon of the algorithm in the edge detection process. The edge detection result of the pixel value calculation function in the image could be expressed as follows:

(10) $$\begin{array}{c} U^{\prime }\left(x,y\right)=\left\{\begin{array}{c} \begin{array}{cc} 0, & R\left(x,y\right)<t_{l}, \end{array}\\ \begin{array}{cc} 1, & t_{l}\leq R\left(x,y\right)\leq t_{h}, \end{array}\\ \begin{array}{cc} q, & R\left(x,y\right)\geq t_{h}. \end{array} \end{array}\right) \end{array}$$

In the equation above, *R*′(*x*, *y*) represented the pixel value in the result image of edge detection, *R*(*x*, *y*) represented the pixel value in the nonmaximum suppression image, and *t*_*l*_ and *t*_*h*_ referred to the low threshold and the high threshold, respectively. When the pixel was adjacent to the edge pixel, *q* was 1; or otherwise it was 0. The Canny algorithm can detect the local edge of the input image, but it was difficult to extract the region of interest in the image. Therefore, after using the Canny algorithm to extract the edge points of the image, the PSO algorithm was adopted to extract the features of the WJI patient MRI image edge and optimize the penalty factor and kernel parameters of the SVM algorithm. When the PSO algorithm was adopted to extract edge features, it was assumed that a particle swarm of particles was initialized in a multidimensional search space, then the position information of the particles can be expressed by the following equation:

(11) $$\begin{array}{c} H=\left(H_{1},H_{2},H_{3},\ldots ,H_{z}\right). \end{array}$$

In the particle swarm *H*, the position of each particle corresponded to a *n*-dimensional vector, and the position information of the *m*th particle in the *n*-dimensional space can be expressed by the following equation:

(12) $$\begin{array}{c} H_{m}={\left(H_{m1},H_{m2},H_{m3},\ldots ,H_{mn}\right)}^{T}. \end{array}$$

Similarly, the velocity of each particle corresponded to a *n*-dimensional vector, and the velocity of the *m*th particle in the dimensional space can be expressed by

(13) $$\begin{array}{c} V_{m}={\left(V_{m1},V_{m2},V_{m3},\ldots ,V_{mn}\right)}^{T}. \end{array}$$

In the PSO algorithm, each particle had to be performed with the iterative optimization to approach the position of the global optimal solution. In the optimization process, the historical best position and the best position of the group that each particle passes can be expressed by the following equation:

(14) $$\begin{array}{c} p{\text{best}}_{m}={\left(p{\text{best}}_{m1},p{\text{best}}_{m2},p{\text{best}}_{m3},\ldots ,p{\text{best}}_{mn}\right)}^{T},\\ g{\text{best}}_{g}={\left(p{\text{best}}_{g1},p{\text{best}}_{g2},p{\text{best}}_{g3},\ldots ,p{\text{best}}_{gn}\right)}^{T}. \end{array}$$

The iterative equation of velocity and position of particle *m* at time *t* *+* 1 in this algorithm can be expressed by the following equations:

(15) $$\begin{array}{c} V_{mn}\left(t+1\right)=V_{mn}\left(t\right)+\varpi_{1}\rho_{1}\left[p{\text{best}}_{mn}\left(t\right)-H_{mn}\left(t\right)\right]+\varpi_{2}\rho_{2}\left[g{\text{best}}_{gn}\left(t\right)-H_{mn}\left(t\right)\right],\\ H_{mn}\left(t+1\right)=H_{mn}\left(t\right)+V_{mn}\left(t+1\right). \end{array}$$

Here, *V*_*mn*_(*t*+1) represented the *n*-dimensional velocity of the *m*th particle at time *t* + 1; *ϖ*_1_ and *ϖ*_2_ represented the cognitive factor and social factor, respectively; *ρ*_1_ and *ρ*_2_ were random numbers that obey the normal distribution, and the value range was [0,1]; *p*best_*mn*_(*t*) was the best position in the *n*th dimension of the *m*th particle at time *t*; *g*best_*gn*_(*t*) referred to the best position in the *n*th dimension of all particles at time *t*; and *H*_*mn*_(*t*+1) represented the position of the *m*th particle in the *n*th dimension at time *t* + 1.

**Figure 1:**
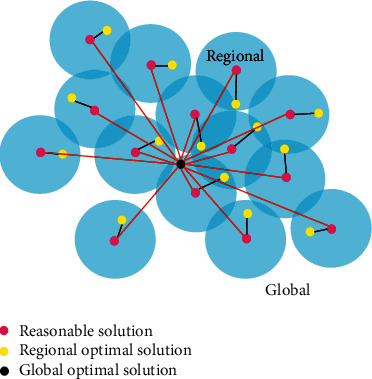
Optimization of PSO algorithm.

The optimization process of PSO algorithm is shown in Figure 1.

The PSO algorithm showed small data volume, simple operation, and high computational efficiency, but it was easy to fall into the local optimal solution in the later stage of optimization. Therefore, the SVM algorithm was adopted to obtain the global optimal solution under limited information. It was assumed that there was a data set (*a*_*i*_, *b*_*i*_) (*i*=1,2,…, *j*). In which, *j* referred to the number of training samples in the data set, *a*_*i*_ represented the features vector of the data set, and *b*_*i*_ referred to the category of the data set. Then, the decision boundary of an image can be expressed by the following function:

(16) $$\begin{array}{c} f\left(a\right)=\left(u\cdot a\right)+ο=0. \end{array}$$

In equation (16), *u* and *O* represented the weight vector and the offset, respectively. *F* was denoted to be the separating hyperplane; *F′* and *F″* were denoted the edge hyperplanes; and the vectors on *F′* and *F″* were collectively called support vectors. In general, the distances from *F* to the support vectors on the edge hyperplanes were equal, which can be expressed by the following function:

(17) $$\begin{array}{c} \left\|u\right\|=\sqrt{u_{1}^{2}+u_{2}^{2}+\cdots +u_{c}^{2}}. \end{array}$$

The parameters *u* and *O* had to be calculated to calculate *F*, which was minimized to :

(18) $$\begin{array}{c} S\left(u\right)=\frac{{\left\|u\right\|}^{2}}{2}. \end{array}$$

The constraints were defined as follows:

(19) $$\begin{array}{c} g_{i}\left[\left(u\cdot a_{i}\right)+ο\right]\geq 1,\;i=1,2,\ldots ,j. \end{array}$$

The Lagrangian function was introduced to solve the constrained optimization of the SVM algorithm:

(20) $$\begin{array}{c} L=\frac{{\left\|u\right\|}^{2}}{2}-\sum_{i=1}^{j}\kappa_{i}g_{i}\left[\left(u\cdot a_{i}\right)+ο\right]+\sum_{i=1}^{j}\kappa_{i}. \end{array}$$

In the above equation, *L* represented the Lagrangian function; *κ*_*i*_ represented the Lagrangian coefficient, and the solution of *L* was obtained by taking the partial derivatives of *u* and *O*, which was set to 0. Thus, the above problem can be transferred into a dual problem, which was maximized as follows:

(21) $$\begin{array}{c} U\left(\kappa \right)=\sum_{i=1}^{j}\kappa_{i}-\frac{1}{2}\sum_{s,i=1}^{j}\kappa_{i}\kappa_{s}g_{i}g_{s}\left(a_{i},a_{s}\right). \end{array}$$

The constraints were defined as follows:

(22) $$\begin{array}{c} \left\{\begin{array}{c} g_{i}\left[\left(u\cdot a_{i}\right)+ο\right]\geq 1,\\ \sum_{i=1}^{j}\kappa_{i}g_{i}=0,\\ \kappa_{i}\geq 0,i=1,2,\ldots ,j. \end{array}\right) \end{array}$$

According to the above equation, the optimal solution about the Lagrangian coefficient was obtained:

(23) $$\begin{array}{c} \kappa^{*}=\left(\kappa_{1}^{*},\kappa_{2}^{*},\ldots ,\kappa_{j}^{*}\right). \end{array}$$

The optimal weight and the optimal offset were expressed as the following equations:

(24) $$\begin{array}{c} u^{*}=\sum_{i=1}^{j}\kappa_{i}g_{i}a_{i}, \end{array}$$

(25) $$\begin{array}{c} {ο}^{*}=g-\sum_{i=1}^{j}\kappa_{i}g_{i}\left(a_{i},a\right). \end{array}$$

In equations (24) and (25), *κ*_*i*_ was 0 in most cases. When *κ*_*i*_ ≠ 0, the sample vector that held the constraints in equation (19) was used as the support vector to obtain the optimal segmentation hyperplane and optimal decision function expression The equation was as follows:

(26) $$\begin{array}{c} f\left(a\right)=\mathrm{sgn}\left\{\left(u^{*}\cdot a\right)+{ο}^{*}\right\}=\mathrm{sgn}\left\{\sum_{i=1}^{j}\kappa_{i}^{*}g_{i}\left(a_{i},a\right)+{ο}^{*}\right\}. \end{array}$$

When the optimal decision function was calculated, only the support vector had to be summed, and the support vector at *κ*_*i*_ ≠ 0 determined the classification result of the algorithm. In the face of linear inseparability, the SVM algorithm would map the input vector to the high-dimensional vector space to find the separation hyperplane. At this time, the nonlinear separable kernel function and the optimal decision function can be expressed as below equations:

(27) $$\begin{array}{c} K\left(a_{i},a\right)=\left[\gamma \left(a_{i}\right)\cdot \gamma \left(a\right)\right], \end{array}$$

(28) $$\begin{array}{c} f\left(a\right)=\mathrm{sgn}\left\{\left[u^{*}\cdot \gamma \left(a\right)\right]+{ο}^{*}\right\}=\mathrm{sgn}\left\{\sum_{i=1}^{j}\kappa_{i}^{*}g_{i}\left[\gamma \left(a_{i}\right)\cdot \gamma \left(a\right)\right]+{ο}^{*}\right\}. \end{array}$$

**Figure 2:**
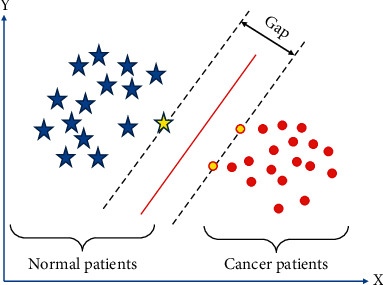
Classifications of linearly separable SVM.

**Figure 3:**
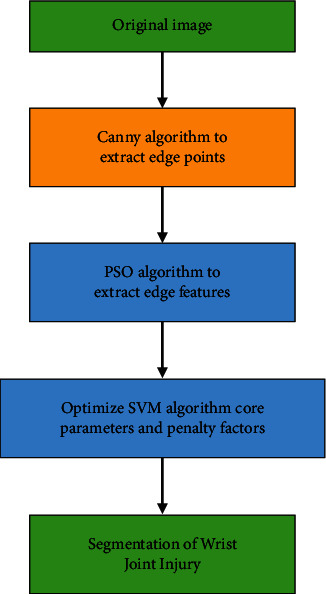
Overall flow chart of this thesis.

In equations (28) and (29), *K*(*a*_*i*_, *a*) represented the kernel function and *γ* represented the nonlinear mapping. When the contour of the injured part on the WJI patient's MRI image was segmented, the Canny algorithm was first used to extract the thick edges with relatively strong pixel differences in the image. In order to avoid region growth leading to inaccurate segmentation, the PSO-SVM algorithm as applied to classify the image edges. The PSO algorithm was adopted to select the features of the MRI image edge and then the kernel parameters and penalty factors of the SVM algorithm were optimized, realizing the accurate identification and segmentation of the WJI part.  Figure 2 illustrated the classifications of linearly separable SVM (Figure 3).

### 2.4. Image Processing Quality Evaluation

For the MRI image segmentation effect of all WJI patients participating in this study, subjective evaluation and objective evaluation were used. The main indicators of subjective evaluation were peak signal to noise ratio (PSNR), mean square error (MSE), figure of merit (FOM), and structural similarity (SSIM). The functional expressions of the above indicators were as follows:

(29) $$\begin{array}{c} \text{PSNR}=10\mathrm{lg}\left[\frac{pe_{\max}^{2}}{1/\left(\hat{h}\cdot \hat{l}\right)\sum_{x=1,y=1}^{\hat{h},\hat{l}}{\left[f\left(x,y\right)-\hat{f}\left(x,y\right)\right]}^{2}}\right], \end{array}$$

(30) $$\begin{array}{c} \text{MSE}=\frac{1}{\hat{h}\cdot \hat{l}}\sum_{x=1,y=1}^{\hat{h},\hat{l}}{\left[f\left(x,y\right)-\hat{f}\left(x,y\right)\right]}^{2}, \end{array}$$

(31) $$\begin{array}{c} \text{FOM}=\frac{1}{\max\left\{\hat{X},X_{\text{ideal}}\right\}}\sum_{\hat{c}=1}^{\hat{X}}\frac{1}{1+{\hat{d}}_{\hat{c}}^{2}\rho }, \end{array}$$

(32) $$\begin{array}{c} \text{SSIM}\left(\hat{P},\hat{P^{\prime }}\right)={\left[bri\left(\hat{P},\hat{P^{\prime }}\right)\right]}^{\alpha }\times {\left[con\left(\hat{P},\hat{P^{\prime }}\right)\right]}^{\beta }\times {\left[str\left(\hat{P},\hat{P^{\prime }}\right)\right]}^{\gamma }. \end{array}$$

In equations (29)–(32), *pe*_max_ represented the maximum value of all pixels on the image; $\hat{h},\hat{l}$ represented the height and width of the image, respectively; *f*(*x,y*) and $\hat{f}\left(x,y\right)$ referred to the gray value of each pixel of the image without noise and that of the image after noise reduction, respectively; $\Delta f,\bar{\Delta }\hat{f}$ represented the results of edge extraction of the original image *f* and the denoised image $\hat{f}$ by the 3 × 3 Laplacian operator, respectively; $\hat{X}$ and *X*_ideal_ represented the number of boundary points after image processing and that of ideal image boundary points, respectively; ${\hat{d}}_{\hat{c}}$ was the distance from the $\hat{c}$ image after noise reduction to the closest boundary point; $\hat{P},\hat{P^{\prime }}$ represented the original ideal noise-free image and the noise-reduced image, respectively; and $bri\left(\hat{P},\hat{P^{\prime }}\right)$, $con\left(\hat{P},\hat{P^{\prime }}\right)$, and $str\left(\hat{P},\hat{P^{\prime }}\right)$ represented the brightness, contrast, and structure, respectively.

### 2.5. Edge Continuity and Edge Credibility Detection

Edge continuity and edge credibility are two important evaluation indicators. Whether the edge is continuous is directly related to whether the extraction of the target area is complete. The calculation equation can be expressed as follows:

(33) $$\begin{array}{c} CI\;dx=\frac{\sum_{i=1}^{C}\left(n_{i}\times S\left(E\left(i\right)\right)\right)}{\sum_{i=1}^{C}n_{i}}. \end{array}$$

In the above equation, the value range of *CI*  *dx* is [0,1), and the size of the edge continuity index directly determines the continuity of the image edge.

The calculation equation of the marginal credibility index can be expressed as follows:

(34) $$\begin{array}{c} BI\;dx=\frac{1}{n}\sum_{E\left(i,l\right)=1}b\left(i,l\right). \end{array}$$

In the above equation, *b*(*i*, *l*) represents the processed image, and *n* represents the number of edges. The greater the credibility of the edge, the closer the image is to the real edge.

### 2.6. Observation Indicators

The manual segmentation results of clinically experienced orthopedic surgeons were undertaken as the gold standard to analyze the accuracy, sensitivity, specificity, and Dice similarity coefficient of PSO-SVM algorithm in diagnosis of WJI. The calculation equation was as follows:

(35) $$\begin{array}{c} \text{Acc}=\frac{TP+TN}{TP+TN+FP+FN}, \end{array}$$

(36) $$\begin{array}{c} \text{Sen}=\frac{TP}{TP+FN}, \end{array}$$

(37) $$\begin{array}{c} \text{Spe}=\frac{TN}{TN+FP}, \end{array}$$

(38) $$\begin{array}{c} \text{DSC}=\frac{2\times TP}{2\times TP+FN+FP}. \end{array}$$

In equations (35)–(38), Acc, Sen, Spe, and DSC represented accuracy, sensitivity, specificity, and Dice similarity coefficient, respectively; *TP* represented the number of positive samples that were correctly classified; and *TN* represented the number of negative samples that were correctly classified. *FP* represented the number of negative samples with misclassification, and *FN* referred to the number of positive samples with misclassification.

### 2.7. Statistical Analysis

In this study, SPSS 22.0 software was used for data processing. Measurement data were expressed as mean ± standard deviation, and measurement data were expressed as %. The comparison between groups was performed by SNK-*q* test. *P* < 0.5 indicated that the difference was statistically significant.

## 3. Results

### 3.1. Edge Point Extraction of MRI Image Based on Canny Operator

**Figure 4:**
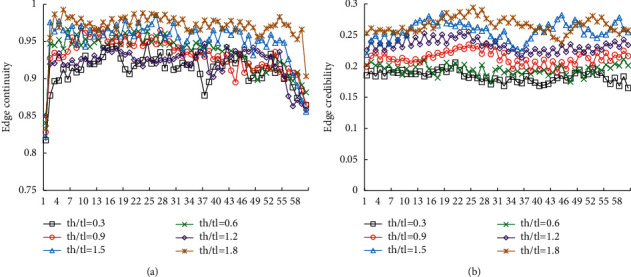
The difference between edge continuity and edge credibility of different scale coefficients. (a) Edge continuity. (b) Edge credibility.

In this study, the Canny algorithm was adopted to extract the edge points of the image, and the ratio coefficient (th/tl) of its high and low thresholds determined the edge detection effect of the algorithm. Therefore, th/tl was modified, and th/tl was set to = (0.3, 0.6, 0.9, 1.2, 1.5, 1.8) to observe the image edge detection results under different iteration scale factors. The detection results were shown in Figure 4. The experimental results showed that when th/tl = 1.5 and 1.8, the test results were similar, but the reliability of th/tl = 1.8 was higher than that at th/tl = 1.5. Therefore, the Canny algorithm's edge detection was the best at th/tl = 1.8.

**Figure 5:**
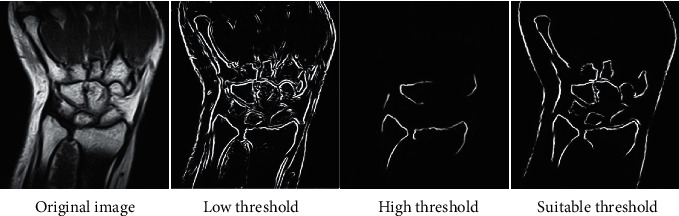
The edge extraction results of Canny algorithm.

After the Canny algorithm scale factor was adjusted to 1.8, edge features were extracted from the WJI patient's MRI image. The results (Figure 5) showed that the patient's MRI image was disturbed by noise and was affected by uneven grayscale. The patient's cartilage tissue was crescent-shaped and thinner, resulting in similar grayscale ranges among the radius, scaphoid, and lunate. The patient's MRI image showed the characteristics of vagueness and complexity. The Canny algorithm was used to extract image edges. When the threshold was too high, there was a serious edge loss in the extraction result. If the threshold was too low, it showed a large number of detected edges. After the th/tl was set to = 1.8, the algorithm showed a better edge extraction effect, but it was difficult to extract the region of interest in the image.

### 3.2. Edge Segmentation Result of MRI Image Based on PSO-SVM Algorithm

**Figure 6:**
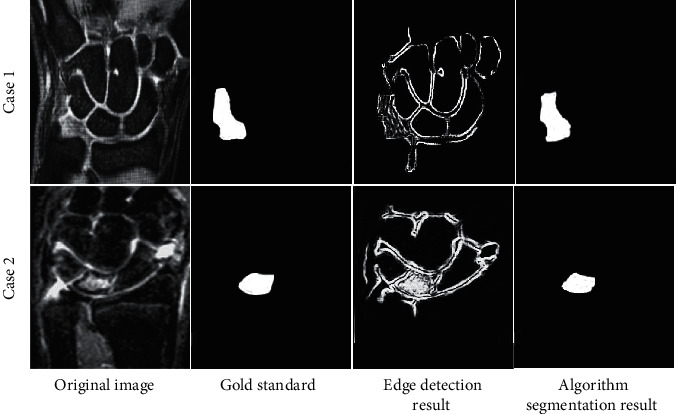
Schematic diagram of the segmentation results of the PSO-SVM algorithm.

The algorithm training was performed on the MRI images of patients participating in this study, and the edge extraction results were shown in Figure 6. Patient 1 was a 45-year-old male. MRI showed that the patient's scapholunate interosseous ligament was interrupted, and there was a synovial cyst in the triangle and ulna. Patient 2 was a 54-year-old female. MRI showed that the lunar bone collapsed and the scapho-moon separation was seen. The results showed that the PSO-SVM algorithm can accurately classify the patient's lesions.

**Figure 7:**
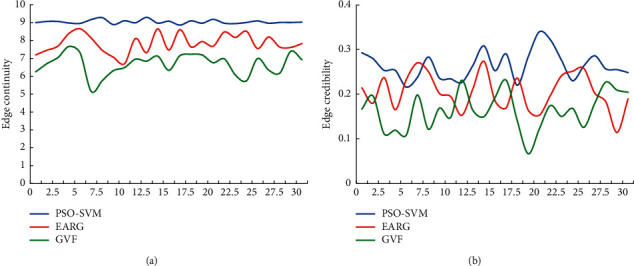
The difference between edge continuity and edge credibility of different algorithms. (a) Edge continuity. (b) Edge credibility.

The mean ± standard deviation was adopted to evaluate the overall level of the image segmentation results, and the GVF and EARG algorithms were introduced for comparison, so as to avoid the contingency of the edge detection results of the PSO-SVM algorithm. The results shown in Figure 7 suggested that the edge continuity of the image after PSO-SVM algorithm segmentation was stable above 0.9, and the edge credibility was stable above 0.2, showing statistically great differences in contrast to other two algorithms (*P* < 0.05).

### 3.3. Comparison of Image Processing Quality Based on Different Algorithms

**Figure 8:**
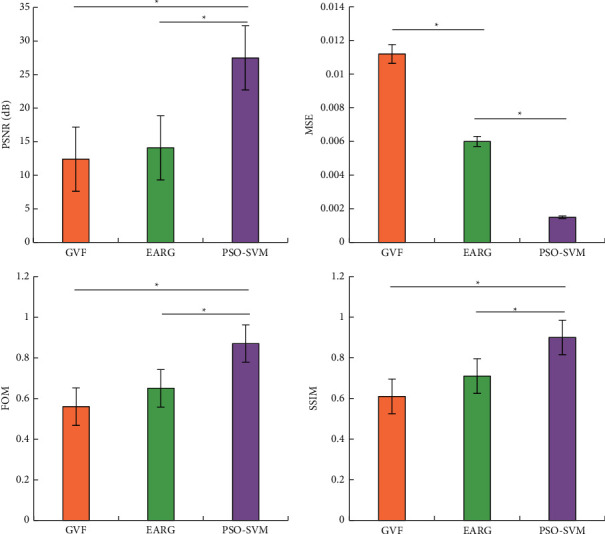
The objective evaluation of different algorithms on the effect of MRI image processing in patients with WJI. ^*∗*^indicated that the difference was statistically obvious.

The PSNR, MSE, FOM, and SSIM were adopted to quantitatively objectively evaluate the processing effect of the PSO-SVM algorithm on the MRI image. The results given in Figure 8 showed that the PSNR, MSE, FOM, and SSIM of the PSO-SVM algorithm were 26.891 ± 5.331 dB, 0.0014 ± 0.0003, 0.8832 ± 0.0957, and 0.9032 ± 0.0807, respectively. The PSNR, FOM, and SSIM values of the PSO-SVM algorithm were much higher than the GVF algorithm and the EARG algorithm, while the MSE value was much lower than the GVF algorithm and the EARG algorithm, and the difference was statistically significant (*P* < 0.05).

### 3.4. Evaluation on Diagnosis Effects Based on Different Algorithms

**Figure 9:**
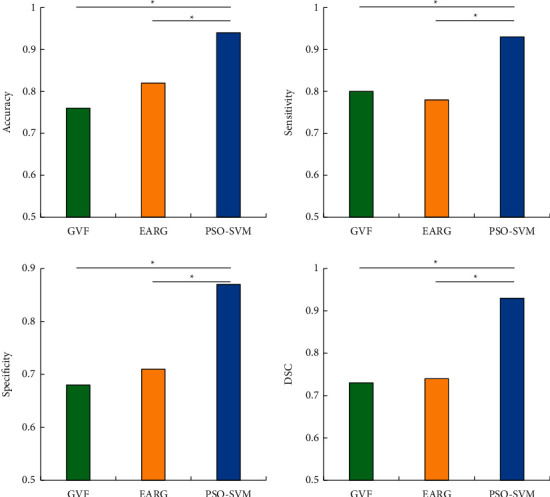
Diagnosis results of different algorithms. *Note.*^*∗*^indicated that the difference was statistically significant.

The pathological diagnosis showed that there were 48 cases of occult fractures, 67 cases of displaced fractures, and 45 cases of dislocations. The accuracy, sensitivity, specificity, and Dice similarity coefficient of the three groups were compared, and the results were shown in Figure 9. The results revealed that the diagnostic accuracy, sensitivity, specificity, and Dice similarity coefficient of the PSO-SVM algorithm were 0.9413, 0.9129, 0.9088, and 0.8715, respectively. The above four indicators were obviously higher than those of the GVF algorithm and the EARG algorithm, and the differences were statistically great (*P* < 0.05).

## 4. Discussion

The wrist joint is formed by intertwining ligaments and attaching each carpal bone. It is frequently used in daily life, so it is easily damaged. If the treatment is improper, it is very easy to cause delayed WJI healing, avascular necrosis of the wrist, joint instability, and even deformity of the wrist joint in severe cases [22]. At present, the clinical diagnosis of WJI is mainly based on imaging examinations, but it is still difficult to recognize in the early imaging examinations after injury due to the complexity of the local anatomy of the wrist [23].

In this study, the Canny algorithm was used to extract the edge of the image, and the edge detection effect of the Canny algorithm was detected through the scale coefficients of high and low thresholds. The results indicated that when the scale factor was 1.8, the Canny algorithm showed the highest edge continuity and edge credibility. Under the high threshold, the Canny algorithm showed serious edge loss during extraction, while under the low threshold, there were too many detected edges. After the scale factor was adjusted to 1.8, the Canny algorithm showed the best edge extraction effect, but it was still difficult to extract the lesion area in the image. Regarding the threshold of the Canny edge detection algorithm, Parthasarathy et al.'s research on MRI images of brain tumors also reached similar conclusions [24]. Therefore, after application of the Canny algorithm, the PSO-SVM algorithm was adopted to further segment the patient's MRI image features. The results disclosed that the PSO-SVM algorithm showed the stable edge continuity stability higher than 0.9 and stable edge credibility higher than 0.2, indicating that the PSO-SVM algorithm showed better effect in contrast to GVF algorithm and EARG algorithm. The objective evaluation revealed that the four indicators of the PSO-SVM algorithm (PSNR, MSE, FOM, and SSIM) were obviously higher than those of the GVF algorithm and the EARG algorithm, and the differences were statistically great (*P* < 0.05). Such results suggested the PSO-SVM algorithm showed better image processing effect. For the diagnosis results of WJI, it was found that this algorithm showed higher diagnostic accuracy, sensitivity, specificity, and Dice similarity coefficient than the GVF algorithm and the ERG algorithm, which suggested that the PSO-SVM algorithm realized higher diagnostic accuracy for WJI.

## 5. Conclusion

In this study, the Canny algorithm was used to extract the edge features of the WJI patient's MRI image and then the PSO-SVM algorithm was used to classify the extracted edges, achieving an excellent edge detection effect. The PSO-SVM algorithm showed higher edge continuity and credibility, better segmentation performance, and higher diagnostic accuracy for WJI. This algorithm not only greatly improved the efficiency of segmentation, reduced a large number of manual repetitive operations, and can assist clinicians in the clinical auxiliary diagnosis of WJI, so it showed high theoretical and practical significance. However, there were still some shortcomings in this study. The structure of the wrist joint was complex, which resulted in large differences in the positions and features of various parts of the MRI images of different patients. Therefore, the running time of the algorithm was long. In addition, it failed to analyze the auxiliary diagnosis effect of the algorithm on WJI. Such shortcomings had to be improved in the follow-up work.

## Data Availability

The data used to support the findings of this study are available from the corresponding author upon request.
